# Practice Recommendations for Resistance Exercise in the Acute Phase After Burns: State of the Science

**DOI:** 10.3390/ebj7040054

**Published:** 2026-10-20

**Authors:** Tyler Jerome Osborne, Samuel E. Ibison, Fiona M. Wood, Brad Wall, David R. Schieffelers, Ulrike Van Daele, Marianne K. Nieuwenhuis, Craig Porter, Dale W. Edgar

**Affiliations:** 1 School of Medical and Health Sciences, Edith Cowan University, Joondalup, WA 6027, Australia; 2 Fiona Wood Foundation, Murdoch, Perth, WA 6150, Australia; 3 State Adult Burn Unit, Fiona Stanley Hospital, South Metropolitan Health Service, Murdoch, Perth, WA 6150, Australia; 4 Burn Injury Research Unit, Faculty of Medicine and Dentistry, University of Western Australia, Crawley, Perth, WA 6009, Australia; 5 School of Allied Health, Murdoch University, Perth, WA 6150, Australia; 6 Research Group MOVANT (Movement Antwerp), Department of Rehabilitation Sciences and Physiotherapy, University of Antwerp, 2000 Antwerp, Belgiumulrike.vandaele@uantwerpen.be (U.V.D.); 7 Faculty of Health, Amsterdam University of Health Sciences, 1081 BT Amsterdam, The Netherlands; 8 OSCARE, (Organisation for Burns, Scar Aftercare and Research), 2000 Antwerp, Belgium; 9 Alliance of Dutch Burn Care (ADBC), Burn Center, Martini Hospital, 9728 NT Groningen, The Netherlands; nieuwemk@mzh.nl; 10 Department for Human Movement Sciences, University Medical Center Groningen, University of Groningen, 9728 NT Groningen, The Netherlands; 11 Research Group Healthy Ageing, Allied Health Care and Nursing, Hanze University of Applied Sciences, 9728 NT Groningen, The Netherlands; 12 Department of Pediatrics, University of Arkansas for Medical Sciences, Little Rock, AR 72202, USA; 13 Arkansas Children’s Research Institute, Little Rock, AR 72202, USA; 14 Institute for Health Research, Burn Injury Research Node, The University of Notre Dame Australia, Fremantle, WA 6160, Australia

**Keywords:** burn injury, acute inpatient rehabilitation, resistance training, muscle atrophy, exercise prescription, clinical practice guidelines

## Abstract

**Highlights:**

**What are the main findings?**

- Early resistance training after burn injury is feasible, safe and may attenuate muscle loss.
- Exercise prescription should be individualised using burn-specific factors and objective intensity monitoring.

**What are the implications of the main findings?**

- Early resistance training may improve long-term exercise adherence.
- Robust trials with patient-centred outcomes are needed.

**Abstract:**

Background: Resistance exercise is a cornerstone of rehabilitation after burn; however prescription of resistance-based exercise during acute adult burn recovery remains empiric and experience-based, varying widely between centres. To understand the contemporary evidence available to guide strength training prescription acutely after burn injury, we conducted a narrative review. Methods: PubMed, PEDro and the supplementary literature were searched for English, Dutch, French and German language human studies evaluating structured resistance training in adult burn populations. Studies eligible for inclusion were: (1) those conducted on humans (no animal or in vitro studies were included); (2) those that investigated the effects of a structured resistance exercise intervention initiated acutely after burn; and (3) those including combined aerobic exercises. The key practical and clinical considerations were timing, dose, monitoring, safety, and outcome selection. Results: After screening, a total of three full-text studies were reviewed. High-quality trials were scarce: three randomised controlled trials implemented structured resistance exercise during the inpatient phase. Study protocols, burn severity, and endpoints were heterogeneous and many functional and quality-of-life outcomes were underpowered. Discussion: There is evidence from multiple independent studies that early, supervised resistance-focused training commenced acutely post-injury is feasible and generally well tolerated, with positive signals for attenuation of muscle wasting and improvements in functional outcomes. Although the evidence base remains limited, the consistency of findings across available studies conducted in different settings and populations increases confidence in these recommendations presented herein. Conclusion: Combining available evidence and reflecting unique, burn-specific constraints, this review presents a revised framework for prescription of resistance training, emphasising individualisation and flexibility and session-by-session progression guided by clinical status and perceived exertion.

## 1. Background

The primary objective of resistance exercise after injury is to restore, and potentially enhance, an individual’s muscle mass, functional physical strength and independence to pre-injury levels, with a secondary objective of averting long-term complications, including insulin resistance and other internal sequelae [1]. Resistance exercise practices vary considerably between burn centres, and standardised, evidence-based guidelines are currently lacking, yet there is guidance available for the prescription of load, volume and frequency of training in healthy and other populations [2]. The existing literature supports resistance exercise as a beneficial post-burn recovery strategy—contributing to the preservation of physical function and improvements in health-related quality of life (QoL) [3,4,5,6,7,8]. Post-burn resistance exercise prescription is structured with shifting priorities of focus around the sliding milestones of recovery, initial stabilisation, rehabilitation, and longer-term functional restoration. Importantly, while early initiation of resistance exercise is critical for counteracting the profound catabolic and metabolic disturbances that emerge immediately after burn injury, the benefits of exercise participation are not confined to the acute phase. Rather, engagement in resistance exercise during rehabilitation may provide an important opportunity to establish sustainable physical activity behaviours and promote broader healthy lifestyle habits. Where appropriate, clinicians should view early exercise participation as a foundation for long-term engagement in evidence-based health and lifestyle behaviours that support metabolic health, functional capacity, and protection against chronic disease throughout recovery and beyond. It is also acknowledged that it is impossible to isolate and attribute the effects of exercise treatments after burn to resistance training alone, as aerobic exercise is commonly incorporated as a warm-up, cool-down, or concurrent component of rehabilitation programmes. Given the frequent co-administration of aerobic and resistance training in the included studies, the relative contribution of each modality to the reported outcomes cannot be determined from the current evidence. Nevertheless, resistance training remains particularly relevant to the primary muscle mass-related impacts and impairments observed following burn injury. The post-burn period is characterised by “targeted” muscle catabolism, losses in lean mass and strength, and consequent reductions in physical function [9], all of which are directly targeted by resistance exercise. Accordingly, the focus of this review was to evaluate the evidence supporting resistance-focused rehabilitation and its role in addressing these key impairments. While the available studies often incorporated additional exercise components, the evidence consistently supports the feasibility and tolerability of early resistance-based interventions, with favourable signals for preserving muscle mass, improving strength, and enhancing physical function following burn injury. Resistance training therefore has a strong theoretical rationale for addressing the primary catabolic and functional consequences of burn injury [10,11]; however, direct comparative trials of resistance-only arms versus aerobic-only and combined resistance and aerobic interventions are needed to establish the relative contribution of each component. This represents an important area for future research but falls beyond the scope of the current review.

In the early after-burn phase, resistance exercise interventions primarily target oedema management, benefiting from the muscle pump and attenuation of muscle unloading and wasting via stimulation of muscle contractions. As the patient recovers, the focus of the goal progresses to that of more traditional resistance training and range of motion restoration, or maintenance, to optimise recovery during scar maturation and restore long-term functional capacity. Resistance exercise-based interventions have been shown to reduce muscle wasting and insulin resistance across healthy, diabetic, and critically ill populations, with emerging burn research—mostly in paediatric and post-discharge settings—indicating similar metabolic benefits in terms of the restoration of lean body mass, muscle strength and cardiorespiratory fitness [12]. Yet the use of resistance exercise to target metabolic outcomes during adult inpatient burn care remains largely unexplored, and current practice inconsistently incorporates metabolic parameters and inflammatory responses as therapeutic targets, highlighting the need for clearer guidance [13].

Acute burn injury triggers a profound hypermetabolic and catabolic response characterised by accelerated muscle wasting and stress-induced disturbances in glucose regulation. These systemic challenges create a physiologically fragile baseline from which patients must attempt to regain function, underscoring the importance—and difficulty—of early, targeted rehabilitation. Collectively, the progressive physiological adaptations achieved through resistance exercise support patient-centred goals of returning to pre-injury participation, social functioning, and work productivity, and contribute to recovery of multidimensional QoL. However, daily fluctuations in clinical status are a hallmark of acute burn recovery, creating a unique challenge in applying traditional prescription parameters and progression strategies [3]. Shifts in pain, wound condition, metabolic demand, and overall stability can alter a patient’s capacity from one session to the next. In addition, events such as surgery impact the validity and customisation of resistance exercise prescription decisions, particularly progression of load. This variability requires resistance exercise prescription to remain highly adaptable, with clinicians adjusting workloads in response to the patient’s presentation on the day, rather than relying on predetermined progressions or past performance.

Despite the potential benefits of empirically determined resistance exercise, the overall quality of evidence to inform clinical strength training prescription for adult patients with burn injury is poor and constrained by methodological heterogeneity and small sample sizes, restricting internal validity and comparability across studies [2]. Given the established benefits of resistance exercise in other clinical populations, such as cancer, where early intervention has been demonstrated to improve recovery trajectories and reduce long-term morbidity [14,15,16], our aim was to conduct a narrative review to inform the key clinical practice considerations for the long-term prescription of resistance exercise in adults commencing early after-burn.

## 2. Methods

This narrative review was designed to identify the current evidence for clinical guidance in prescription of resistance exercise during rehabilitation commencing during the first seven days after burn.

### 2.1. Search Strategy

An online literature search was conducted in PubMed and PEDro (Physiotherapy Evidence Database, a key resource for evidence-based physiotherapy research) to identify studies examining resistance exercise during acute burn recovery. Searches included all articles published up to June 2026. In PubMed, the following search strategy was applied: [(burn OR burns) AND (resistance training OR strength training)]. A supplementary search was conducted in PEDro using the following criteria: therapy = strength training; problem = skin lesion, wound, burn; and method = clinical trial. Searches were restricted to articles published in English, Dutch, French or German due to author language proficiency, and to adult (individuals ≥ 18 years of age) populations because of the distinct physiological and functional characteristics of paediatric burn populations.

This review was conducted as a narrative synthesis of the available literature. Titles and abstracts were independently screened by two authors (TO and SI), with potentially eligible full-text articles subsequently assessed against predefined inclusion and exclusion criteria. Studies were eligible for inclusion if they: (1) were conducted in human participants; (2) investigated a structured resistance exercise intervention initiated acutely post-burn after medical stability was established; and (3) could include a concurrent aerobic exercise component. Studies were excluded if they: (1) were conference abstracts; (2) were review articles, commentaries, or theoretical papers; (3) examined populations outside the acute post-burn phase; or (4) were non-peer-reviewed publications or book chapters. Disagreements regarding study eligibility were resolved through discussion and consensus.

**Figure 1:**
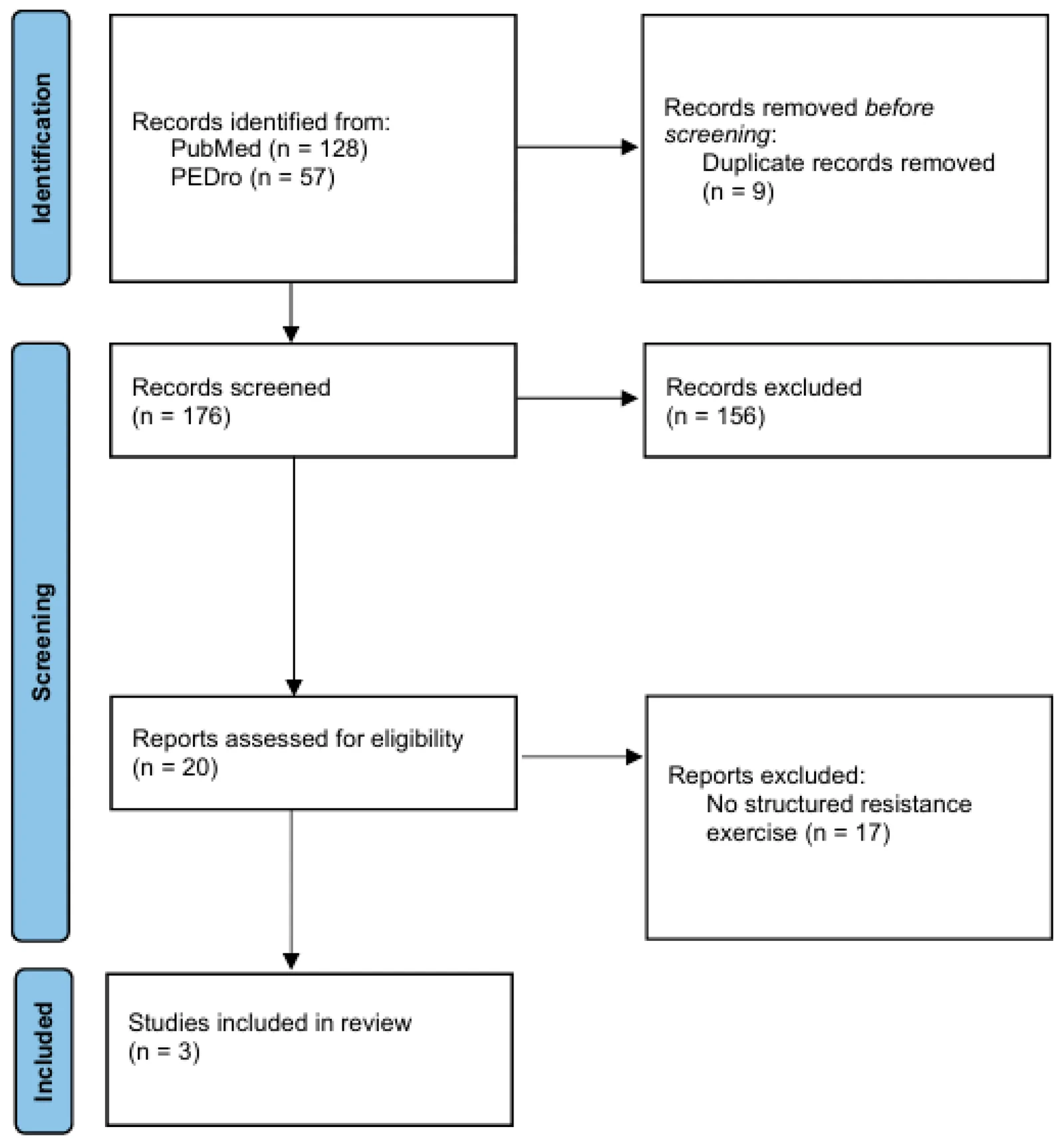
PRISMA flow diagram of the study identification, screening, and selection process.

Given the specific focus on resistance exercise commenced during the acute phase of burn recovery, only three studies met the eligibility criteria for inclusion (Figure 1). Rather than undertaking a formal systematic review or meta-analysis, the available evidence was narratively synthesised with attention to intervention characteristics, implementation feasibility, clinical outcomes and mechanistic findings. To provide a broader physiological and clinical context, additional relevant studies, including mechanistic investigations and exercise research, were identified through supplementary bibliography searches and are discussed throughout the manuscript (Figure 1).

To strengthen the interpretation of the evidence, the certainty of evidence for the included studies was further independently assessed by two authors (TO and SI) applying the Grading of Recommendations Assessment, Development and Evaluation (GRADE) framework [17]. GRADE evaluates the confidence that the estimated effects reflect the true effects by considering the domains of risk of bias, inconsistency, indirectness, imprecision, and publication bias. Randomised controlled trials were initially considered high-certainty evidence but could be downgraded where concerns were identified across these domains. Overall certainty ratings were classified as high, moderate, low, or very low, reflecting the degree of confidence that the available evidence supports the reported findings and subsequent recommendations. These assessments were used to contextualise the strength of conclusions drawn from the narrative synthesis, rather than to formally exclude studies or weight findings quantitatively.

Owing to the limited burn-specific evidence base, recommendations were informed by the corpus of available evidence, including studies from other clinical and healthy populations where physiological principles and exercise responses were considered applicable to burn rehabilitation. This approach was used to contextualise exercise prescription parameters not directly addressed by burn-specific trials.

### 2.2. Clinical Practice Recommendations

When Should Post-Burn Resistance Exercise Be Commenced?

The optimal timing for initiating resistance training after a burn injury varies and should be informed by individual clinical factors, intended time-sensitive goals or outcomes of the rehabilitation programme, burn severity, and grafting status. Gittings et al. [5] demonstrated at their centre that large-muscle-group resistance training was safe to introduce within three days after burn, with pauses of up to three days after acute surgical intervention. Complementing this, findings from a recent multicentre trial in Belgium reinforced that resistance training could be commenced earlier—within one day of admission in more than half of participants—when applying readiness and safety criteria adapted from the ICU population [8]. Importantly, those who did not meet the recruitment criteria at admission were almost exclusively patients requiring mechanical ventilation, underscoring that medical stability, patient alertness, and capacity to cooperate may be more decisive determinants of exercise initiation than injury severity alone [8]. In a study focusing on investigating resistance training in severe burns, participants were able to commence at a median of seven days but ranged from as early as one day post-burn [6], providing evidence that resistance exercise can be safely commenced during the acute inpatient phase regardless of burn size.

Historical evidence indicated that rehabilitative or therapeutic exercise, which commonly incorporates resistance training, should be introduced following discharge from acute care [18] and many programmes begin within one to three months post-burn, with earlier initiation in this window associated with greater improvements in lean body mass in some studies [18,19]. Earlier studies suggested even greater delay in initiation of resistance training at six months to one year post-burn, highlighting the progression from historical decision-making and research in practice [2]. While inpatient exercise is important for mitigating the negative effects of bed rest, the acceptance and widespread deployment of resistance training during the acute phase remains limited and unclear [5,20].

Skin graft integrity is a key consideration when determining when to initiate resistance training. Ambulation and dependency appear safe for individuals with smaller lower-leg grafts who are otherwise medically stable [21,22]. However, in cases involving larger or more complex grafts, earlier initiation of resistance exercise is perceived to pose a risk to graft healing [3]. Considerations regarding graft size and anatomical location in reference to proximity to joints and potential for shear stress with movement are also important. Lower-limb grafts may pose greater challenges when large muscle groups are targeted during exercise sessions to address metabolic disturbances. In contrast, when the graft does not cross a joint, exercise options may be less restricted, allowing earlier and more extensive mobilisation strategies. In patients with severe burns, early resistance training may also be limited by prolonged sedation and reduced capacity for active participation. These potential risks must be balanced against the consequences of immobilisation, as even short periods have been shown to negatively impact physiological and long-term functional outcomes [23]. Further research is required to better define safe and effective timelines for resistance training initiation across different graft presentations and burn severities.

### 2.3. Are There Any Limitations on Resistance Exercise Due to Burn Severity?

Burn severity—which encompasses burn depth and associated likelihood of surgical intervention and systemic disturbances—is a more meaningful determinant of rehabilitation planning than total body surface area (TBSA) alone. In practice, TBSA itself has little direct influence on resistance exercise prescription, as specific limitations are not driven by burn size. Instead, the factors that often accompany severe burns—such as reduced mobility requirements after surgery, sedations, and medical instability—are what constrain early initiation. Although metabolic derangements become more pronounced in individuals with >20% TBSA injuries [19], where hypermetabolism and loss of muscle mass are associated with burn size [24,25], more recent data indicate that smaller burns can also elicit similar systemic metabolic responses [26], reinforcing that TBSA may be an unreliable indicator of exercise tolerance or suitability. These systemic effects may influence the intensity that can be safely prescribed, but they do not inherently preclude resistance training. In severely injured patients, early initiation may be delayed primarily due to sedation or mechanical ventilation rather than burn size. Thus, while burn severity shapes the clinical context, TBSA alone should not be viewed as a limiting factor in determining whether or how resistance exercise can be prescribed.

Burn severity also influences out-of-bed mobility and access to training equipment. Patients with deep or extensive burns and multi-resistant infections may be bed-bound or unable to leave their room, requiring clinicians to adapt exercise prescription to be accessible in the context. In these situations, body weight and low-equipment options, such as resistance bands, small portable weights, or body-weight-assisted exercises, are clinically useful strategies to maintain training stimulus despite mobility restrictions [27]. The challenge with these modes is determining an effective training load, with perceived exertion being the most pragmatic [28].

Another potential limitation to prescription of resistance exercise arises with the dynamic impact of persistent hypermetabolic and immunological healing responses post-burn. In order to balance the catabolic nature of a burn injury [29], nutritional supplementation needs to be carefully considered in order to strike a balance between promoting healing (with the sacrifice of skeletal muscle as a key fuel source) and exercise (building/maintaining muscle) [30]. Indeed, stable isotope data in adults with severe burns suggested that release of, and net loss of, amino acids from skeletal muscle occur alongside marked accretion of amino acids into burn wounds [31]. This underscores the need for careful consideration of the nutritional, particularly protein, needs of burn patients. Further, this highlights the challenges of using current methods to accurately measure protein losses in determining macronutrient prescription in the acute burn healing period.

### 2.4. Does the Location of the Burn Influence Resistance Exercise Prescription?

The anatomical location of a burn injury plays a substantial role in shaping resistance exercise prescription, influencing exercise selection, achievable intensity, and progression strategies. Burn injuries to the hands and feet likely require adapted exercise or reductions in the load that can be prescribed as the body interacts with training equipment or the environment; however, in the study of Gittings et al. [5], even low-load exercise produced meaningful improvements, suggesting that movement itself confers therapeutic benefit. These results provide hope for exercise challenges with the common issue of hand burns. In this context, Gittings et al.’s [5] findings demonstrated the value of clinicians making the effort to adapt the interface or equipment to add load when hand grip is impeded. Importantly, the influence of burn location on prescription also depends on the intended outcome, with resistance training aimed at counteracting and correcting metabolic derangements, potentially requiring different muscle groups and intensity and loading strategies than exercise designed to improve activities of daily living, functional capacity, or quality of life [5]. Burns involving one or more major joints are associated with an increased risk of muscle wasting, stiffness, and contracture formation [32]. Furthermore, delays in initiating movement may limit opportunities for early exercise prescription and contribute to ongoing functional impairment [32]. In paediatric cohorts, early high-intensity exercise has been shown to reduce the need for later surgical release, underscoring the protective role of movement across different therapeutic aims. Findings from the treatment of axillary burns reinforce this, with Webb et al. (2011) investigating the efficacy of splinting the shoulder compared to a control group, who performed mobility and gym-based shoulder exercises [33]. The study found that the exercising control group maintained shoulder function and range of motion (ROM) three months after the axillary burn injury. Burn location may also be a key factor contributing to delays in mobilisation [33]. Patients with burns involving the face, neck, head and upper limbs may restrict the types of exercises that can be safely performed and influence the timing of resistance exercise initiation [34].

### 2.5. Programming Principles for Resistance Exercise After Burn

A dedicated framework for resistance exercise prescription is essential to translate assessment findings into safe, effective and clinically meaningful exercise. In the context of burn rehabilitation, resistance exercise refers to planned, structured exercise performed against an external or body-weight resistance with the intention of improving or maintaining muscle strength, muscle mass, and physical function. This differs from active movement, range-of-motion exercise, muscle activation, positioning, mobilisation, or general therapeutic activity, which may contribute to recovery but are not specifically designed to provide a resistance training stimulus. While existing guidelines outline broad parameters, the acute burn context requires explicit consideration of how load, volume, frequency and progression should be operationalised in practice.

Initial loading should reflect the patient’s current functional capacity, wound status and tolerance to movement. Early sessions often begin with low-to-moderate intensities (~50–70% of three-repetition maximum (3RM) or equivalent isometric force), consistent with previous burn population resistance exercise interventions [5,6,8]. In the earliest phase—particularly for bed-bound or medically fragile patients—load may be derived from bodyweight, resistance bands or gravity-minimised exercises. As patients stabilise, intensities can be progressively increased towards traditional hypertrophy-oriented ranges (~60–80% of 3RM or equivalent isometric force), provided that pain, wound integrity and fatigue remain within acceptable limits [35].

Training volume should be intentionally conservative early on, typically 2 to 3 sets of 8 to 12 repetitions for major muscle groups [35], with exercise selection prioritising large lower limb and trunk musculature due to their relevance for metabolic regulation and functional recovery [36]. For patients with restricted mobility or graft-related limitations, clinicians may employ isometric overflow, modified or partial range movements, unilateral exercise, or low-equipment options such as resistance bands, portable weights, or bed-based bridging and hip-dominant patterns [37,38]. As mobility improves, exercise selection should expand to include multi-joint, functional movements that support gait, transfers, and activities of daily living.

Evidence from early commencement of resistance exercise after burn trials suggests that resistance training performed approximately three times per week is feasible and well-tolerated during acute inpatient care [5,6,8]. Long-term benefits were evident after as few as 10 sessions over four to five weeks [5]. This frequency aligns with general resistance training principles [35] and allows sufficient recovery in the context of heightened metabolic demand, wound healing, and fluctuating clinical status. On days when resistance training is not appropriate, clinicians may substitute with lower-intensity adapted exercises to maintain activity without overloading recovering tissues.

Progression remains a core principle of resistance exercise prescription. However, traditional models based on planned weekly load increments are essentially impractical during acute burn hospitalisation. Pain, wound status, surgical schedules, infection, and fluctuations in functional capacity necessitate considerations for more frequent modification of exercise prescription. Timely decision-making is warranted to achieve the goal of a physiological response or a stimulus to negate muscle degradation during the acute burn period. Consequently, progression should be guided by regular reassessment of patient tolerance, movement quality, perceived exertion, and functional performance rather than adherence to predetermined loading schedules. Improvements may be achieved through increases in external resistance, repetitions completed, range of motion, exercise complexity, or training volume. Equally, temporary reductions or maintenance phases may be required following surgery, grafting, infection, or periods of heightened fatigue.

### 2.6. Is an Assessment Needed to Determine the Starting Point and Intensity of Prescription?

Establishing an appropriate initiation for resistance exercise after burn injury requires a structured assessment, but the acute phase presents unique challenges that limit the utility and validity of fixed baseline measures. A comprehensive evaluation remains essential, beginning with confirmation of medical stability, orthopaedic limitations, oxygen saturation and neurological function [3,8]. Once medically cleared, patients typically undergo exercise-specific assessment to aid in determination of functional capacity and appropriate intensity for resistance training. A 3RM test can be used to estimate initial training intensity, with previous interventions in burn populations commencing at approximately 50–60% of the measured 3RM on any given day [2,39]. Isometric strength testing has also demonstrated utility, with handheld dynamometry being used at the start of each session to monitor strength changes and adjust loads accordingly, though translation into a prescription for load through full joint range of motion is not straightforward [40].

Although these assessments theoretically provide a clear baseline for prescription, their practicality in the acute burn phase may be limited. Daily fluctuations in pain, wound status, fatigue, and overall clinical presentation mean that fixed baseline measures may not effectively capture a patient’s capacity on any given day [3]. As a result, clinicians must interpret assessment data with caution and rely on flexible, session-by-session decision-making rather than rigid adherence to predetermined targets.

### 2.7. Is an Assessment Needed to Guide Ongoing Prescription?

Progression of resistance training similarly requires adaptable monitoring strategies. Rating of perceived exertion (RPE), and particularly sRPE, is widely used to guide progression and has been shown to differentiate between training intensities in post-burn populations [28,40]. Given the pronounced hypermetabolic response following burn injury, traditional physiological markers such as heart rate and blood lactate may be unreliable indicators of exercise intensity. In this context, sRPE offers a valid alternative for regulating training load and ensuring that exercise remains within a safe and tolerable range [28,40]. An earlier systematic review and meta-analysis by Gittings et al. [2] also suggested that physical function, pain, and quality of life are useful measures to assist with guiding prescription [2].

Alongside subjective measures, continuous observation of the patients’ physical and physiological responses remains critical to adjustment of the resistance exercise load and prevention of adverse events. Clinicians monitor breathing patterns, perspiration, movement quality, and signs of symptom exacerbation, recognising that both functional capacity and physiological tolerance can vary markedly from session to session. When patients are unable to meet previously achieved performance levels, exercise intensity must be adjusted accordingly to avoid excessive strain or interference with wound healing [28].

Effective monitoring and progression also depend on coordinated multidisciplinary teamwork and communication. Decisions regarding exercise participation are often informed by input from nursing, medical, physiotherapy, and occupational therapy staff, incorporating factors such as clinical stability, fatigue, oxygen saturation, pain, wound status, and scheduled medical or surgical interventions [41]. Exercise sessions must be integrated into the patient’s broader daily care routine, and without clear communication of post-session responses and collaboration across the care team, implementing and progressing resistance training, particularly during hospitalisation, becomes significantly more challenging [41].

Importantly, individualised exercise prescription must extend beyond physiological readiness alone. A holistic view of the patient, including their goals, preferences, psychological state, and the environmental context in which rehabilitation occurs, is essential for ensuring that resistance training is meaningful, acceptable and sustainable. These factors influence not only sustained engagement and adherence, but also how patients perceive effort, tolerate discomfort, and respond to progression [42].

As acute burn recovery is characterised by substantial daily variability, fixed assessments provide limited guidance for progression. Effective resistance exercise prescription in this phase relies on flexible, responsive clinical reasoning that prioritises the patient’s presentation on the day rather than predetermined benchmarks.

### 2.8. Are Considerations Needed for Long-Term Outcomes and Potential Adverse Events?

Due to the chronic and persistent nature of the post-burn physiology, long-term rehabilitation and exercise are central components for optimal recovery outcomes. Many survivors experience enduring physical, metabolic and functional impairments, which often necessitate extended engagement in structured exercise therapy. Standard rehabilitation programmes typically span 6–12 weeks, with duration frequently tailored to the percentage of TBSA [16]. Ongoing progressive resistance training has been associated with improvements in metabolic, endocrine and cardiovascular outcomes. Positive effects have been observed up to 12–24 months post-injury among those who maintain consistent program adherence [3].

Despite the known benefits of long-term resistance training engagement, clinicians must remain attentive to potential adverse events in the burn context. Overtraining is a particular concern when exercise intensity may not be appropriately monitored or adjusted frequently enough [28]. This may be an issue, as noted above, where a mismatch occurs in the balance between energy and nutrient requirements, and between healing and muscle mass maintenance through resistance exercise. Pain and fatigue are also common factors for early cessation of training and may serve as protective mechanisms against further complications post-burn [28]. Clinicians should bear this in mind when prescribing long-term exercise therapy, with a focus on education around exercise intensity and pain management to improve exercise adherence and avoid premature discontinuation of training sessions.

### 2.9. Outcome Measurement During and Following Resistance Exercise Therapy

Throughout an exercise therapy programme, a range of outcome measurements should be collected to guide clinical decision-making, evaluate patient progress and immediate and post-session responses to the specified prescription. These measures generally fall into two categories: acute measurements—obtained during individual sessions—and longitudinal measurements—obtained periodically throughout the intervention or during long-term follow-up.

Acute measurements typically focus on exercise intensity, workload and immediate physiological responses [40]. Session RPE is a commonly used acute measure that provides real-time insight into a patient’s perceived effort and intensity of the prescribed session. Additional indicators, such as the Hooper index, which incorporates subjective ratings of fatigue, stress, delayed onset muscle soreness and sleep quality, may be administered at the beginning of each session to gauge tolerance over time [28]. However, these parameters have not been validated at any stage of the burn patient recovery journey.

Longitudinal measurements may be used to evaluate the sustained effects of resistance exercise therapy, focusing on physiological function, body composition, and QoL. These outcomes may be assessed months or even years following the intervention [19]. Lean or fat-free mass is a commonly reported long-term measure of body composition and bone mineral content that can be quantified using dual-energy X-ray absorptiometry (DEXA). However, DEXA does not directly measure muscle mass. Alternatives such as bioimpedance spectroscopy for body composition (fat vs. fat-free mass) may be feasible pending validation studies in the post-burn population [43]. Another potential measure to quantify changes to muscle mass is skeletal muscle ultrasound, which Schieffeler et al. [44] demonstrated as a feasible and highly reliable method for quantifying changes in quadriceps muscle architecture throughout acute post-burn care and into later recovery. By providing reproducible assessments of muscle thickness and rectus femoris cross-sectional area—even in the presence of open wounds, oedema, or variable clinical status—it enables objective monitoring of muscle wasting, a phenomenon described in this study with a potentially negative impact on recovery in both the short and long term [44,45].

Strength-related outcomes may be assessed using a variety of tests, including more traditional assessments like 3RM testing, or static tests that are more accessible and easy to administer in hospital settings, including grip strength and isometric strength testing [46]. QoL changes may be assessed using validated patient-reported outcome measures such as the BSHS-B, which captures multiple dimensions of post-burn wellbeing [5,18].

In addition to traditional functional and ROM-based assessments, this framework should also encompass muscle-specific measurements, functional assessments of muscle strength and performance, and biomarkers of muscle catabolism, which together provide a more comprehensive understanding of the patient’s physiological status and response to resistance training [47,48,49].

### 2.10. Review Results

Three studies met the eligibility criteria for inclusion, comprising two randomised controlled trials and one quasi-randomised clinical trial. Certainty of evidence was evaluated using the GRADE framework. Both randomised controlled trials were rated as providing moderate-certainty evidence, reflecting generally robust study designs but with some concerns relating to assessor blinding, attrition, and sample size limitations. The quasi-randomised study was rated as low-certainty evidence due to a higher risk of selection bias arising from non-random allocation procedures and concerns regarding imprecision. Collectively, these findings suggest that, although the evidence base remains small in size, the available studies provide moderate confidence that early exercise interventions confer meaningful benefits during acute burn recovery, particularly in relation to muscle preservation, strength, and functional outcomes. The characteristics and principal findings of the included studies are summarised in Table 1. These studies reinforce that early resistance-focused exercise therapy is a safe and effective adjunct to acute burn care, improving muscle preservation, strength and functional recovery. To the authors’ knowledge, only three studies have implemented resistance exercise interventions commencing during the inpatient phase of burn care, highlighting a critical gap in the evidence base. The single-centre randomised controlled trial (RCT), conducted by Gittings et al. [5], demonstrated that four weeks of resistance training commenced within 72 h of the burn injury was safe and showed short- and long-term benefits for patients who had sustained a burn with a total body surface area (TBSA) ≥ 5% (max TBSA 40%), in comparison to a control group that completed the same exercises with no resistance. In the report by Gittings et al. [5], adverse events were assessed based on two criteria: the need for repeat burn wound surgery and exercise-induced pain. Six of the 38 participants required additional surgical procedures (three in each group). Pain exceeding 7/10 was reported in 57 exercise sessions (15.1% of sessions; 30 in the control group and 27 in the intervention group), though participants requested to stop the session in only nine instances (six in the control group and three in the intervention group) [5]. Importantly, analysis of the Burns Specific Health Scale-Brief (BSHS-B) showed a statistically significant positive weekly effect of the intervention on the Function domain, with the intervention group exhibiting a 21% increase in the odds of recovery relative to the control group (odds ratio 1.21; *p* = 0.017) [5].

A second RCT, conducted by Schieffelers et al. [6], examined the effects of a structured exercise programme initiated approximately one week post-admission in patients with severe burns (40 to 94% TBSA). The authors reported preservation of quadriceps muscle thickness and the rectus femoris cross-sectional area (in the intervention group, corresponding to a 26% greater retention in baseline muscle size over 12 weeks compared with usual care) [6]. While this degree of muscle preservation was interpreted as clinically meaningful—given that post-burn losses of as little as 10% skeletal muscle mass have been associated with impaired wound healing, insulin resistance, and increased infection risk [29]—the functional implications of maintaining a given proportion of skeletal muscle mass remain difficult to interpret in isolation. Nonetheless, the study provides important evidence of a favourable response to early structured exercise, which in that case included both resistance and aerobic components. The early introduction of resistance-focused training is likely a key contributor to attenuating muscle catabolism in the acute phase, even if the precise quantitative impact on long-term function cannot yet be fully established.

Consistent with this, Schieffeler et al.’s [6] study was underpowered to determine a difference in health-related QoL, reflecting a reliance on secondary outcomes rather than pre-specified, adequately powered functional endpoints. This finding aligns with earlier work by Gittings et al. [5], who similarly observed improvements in upper limb function without definitive QoL effects. Together, these studies highlight the challenge of conducting high-quality studies in the acute burn cohort and, further, inferring clinically meaningful functional and QoL benefits from muscle-centric outcomes. Notably no exercise sessions in the Schieffelers et al. [6] study were terminated prematurely due to pain; however, the absence of a predefined pain threshold limits interpretation of intervention tolerability.

A third RCT, also conducted by Schieffelers et al. [8], supports the feasibility and physiological benefits of commencing early structured resistance exercise during acute hospitalisation [8]. In this study, adults with burns ranging from 10 to 70% TBSA commenced resistance and aerobic training as early as possible according to predefined readiness criteria adapted from ICU mobilisation guidelines [50]. These criteria required cardiorespiratory stability (e.g., MAP 60 to 110 mmHg, FIO_2_ < 60%, PaO_2_/FIO_2_ > 200 mmHg, controlled agitation (RASS −2 to +2), normothermia, and lower limb strength ≥ MRC grade 3, with all criteria needing to be met before exercise could begin [50]. More than half of the participants met these criteria immediately upon admission, while those who did not were almost exclusively mechanically ventilated, emphasising that medical stability and patient alertness, rather than burn severity alone, determine readiness for acute resistance training after burn.

Across 412 attempted sessions, 330 were completed successfully, with participants training at a mean RPE of 7.9/10 and without any exercise-related adverse events requiring session termination. The intervention significantly attenuated muscle wasting, with an additional weekly retention of a 0.06 cm quadriceps muscle layer thickness and 0.09 cm^2^ rectus femoris cross-sectional area observed, while quality of life outcomes showed limited between-group differences. Importantly the study demonstrated that acute resistance training can be feasibly and safely integrated into routine inpatient care across multiple centres, even under operational constraints of the COVID-19 pandemic [8].

Taken together, the findings from the three available RCTs provide emerging, multicentre support for the safety and potential efficacy of early, structured resistance training commenced during the inpatient acute burn care period. Collectively, the studies suggest that acute resistance training may play a clinically meaningful role in attenuating severe muscle wasting, supporting functional recovery, and reducing complications associated with early deconditioning after burn. However, the current evidence base remains limited, characterised by a small number of trials, heterogeneity in intervention protocols, burn severity, and outcome measures, and a reliance on secondary or underpowered functional and QoL endpoints.

In light of the profound physiological and functional decline that occurs early following burn injury, and the biological plausibility and emerging clinical evidence supporting resistance training as a countermeasure, this review synthesises the current literature and incorporates additional mechanistic and sentinel studies to inform pragmatic clinical considerations for acute inpatient burn rehabilitation. While acknowledging existing limitations in the evidence base, the findings discussed herein provide a foundation from which cautious, context-specific recommendations may be proposed. These recommendations aim to guide the safe and judicious implementation of acute resistance training, while simultaneously highlighting areas where further high-quality research is required to refine exercise prescription and optimise patient outcomes.

## 3. Discussion

This review highlights the need for more rigorously designed, adequately powered, multicentre randomised controlled trials with diverse sampling, to strengthen the evidence base that informs the development of clinical practice recommendations for adult burn survivor resistance exercise therapy prescription. The most recent guidelines for prescription in the post-burn cohort were published by Rivas et al. (2023) [3]. These guidelines provide a framework that integrates resistance exercise into treatment plans for many conditions, including cancer and diabetes [51,52]. Table 2 below provides the revised burn-context-specific guidelines for clinical prescription of resistance training in adult patients, building on previous research, including the acute recovery period.

### Future Directions

Future research should prioritise trials to clarify the safety, feasibility and efficacy of early commencement of resistance training during the inpatient phase of burn care. To advance the field, future trials should incorporate standardised, phase-specific exercise protocols, clearly define progression criteria, and consistently report adverse events. Drawing on contemporary reporting frameworks such as the Consensus on Exercise Reporting Template (CERT), future trials should provide detailed, replicable descriptions of exercise content, including frequency, load, intensity, duration, supervision, equipment and individualisation, to enable meaningful comparison across studies [53]. Strengthening the methodological transparency in this way will support more robust synthesis, accelerate clinical translation and ultimately refine evidence-based guidelines for acute resistance training after burn.

Equally important is the inclusion of functional and patient-centred endpoints that account for individual variability in pre-injury capacity, age, sex, and comorbidities, enabling more precise interpretation of clinically meaningful change; integrating mechanistic assessments, such as muscle protein turnover, inflammatory markers, and metabolic profiling, may also help to elucidate the biological pathways through which resistance training influences recovery trajectories. Given that hypermetabolism and systemic inflammation after burn contribute to long-term morbidity, including persistent insulin resistance, sarcopenia, and cardiometabolic dysfunction, future trials should explicitly evaluate whether commencing resistance training acutely can mitigate these less visible but clinically consequential sequelae. In parallel, economic outcomes such as length of hospital stay, cost-efficiency, and downstream healthcare utilisation warrant systematic evaluation, particularly in settings where patients face substantial out-of-pocket costs for burn care. Understanding whether early commencement of resistance exercise can reduce resource use or shorten hospitalisation would provide critical evidence for health-system decision-making and improve the accessibility and sustainability of resistance exercise interventions.

A key direction for future work is to better understand how commonly reported barriers to exercise can be addressed rather than interpreted as absolute contraindications. In the study by Schieffelers et al. (2024) [6], most of the identified barriers were modifiable: following graft procedures, exercise can often be adapted rather than discontinued, and challenges related to pain, fatigue, or care routines can be frequently mitigated through patient education, appropriate exercise modification, and coordinated clinical planning. This underscores the central role of multidisciplinary teamwork. Decisions regarding resistance exercise prescription rely on information gathered by multiple healthcare professionals, and effective communication within the care team is essential for safely implementing and progressing resistance training during hospitalisation.

Multisite, large-sample longitudinal research is also needed to determine the sustained impact of early exercise interventions on long-term functional capacity, QoL, and morbidity. Given the chronic physiological consequences of burns, studies extending beyond the typical 6–12-week rehabilitation window are essential to understand how early inpatient resistance training interacts with long-term outpatient or community-based exercise programmes. Future work should also explore patient-centred outcomes [54], including return to work, social participation, and psychological wellbeing, as well as the feasibility of implementing personalised, adaptive exercise models that respond to day-to-day fluctuations in pain, fatigue, and thermoregulatory capacity.

The review was limited to searches of PubMed and PEDro and the inclusion of studies published in four languages. As a result, relevant studies indexed in other databases (e.g., Embase, CINAHL, Scopus or Web of Science) or published in other languages may not have been captured, introducing the potential for publication and language bias.

## 4. Conclusions

The emerging evidence suggests that early, structured resistance exercise therapy during the inpatient phase of burn care can play a meaningful role in mitigating muscle wasting, supporting functional recovery, and enhancing long-term outcomes. Importantly, commencing resistance training early may also help establish the behavioural foundations and confidence required for sustained engagement across the recovery continuum after burn, reinforcing its contribution to long-term physical and metabolic health. This review synthesised the available moderate-quality evidence to inform pragmatic, context-specific considerations for acute resistance exercise prescription, while emphasising the need for further robust research to refine clinical guidelines and customised prescription for burn patients in all care settings.

## Figures and Tables

**Table 1:** Characteristics of the included studies evaluating early resistance exercise after acute burn injury.

Author	Sample Size	Resistance Prescription	Aerobic Exercise Included	Timing of Intervention	TBSA (Median (IQR)) %	Adverse Events
Gitting et al. [5]	Exercise = 23 Control = 25	**Duration:** 4 weeks. **Frequency:** 3 sessions per week. **Intensity:** 70% of daily measured MVIC, 3 sets of 8 to 12 repetitions.	Only as warm-up—5 min exercise bike.	Within 72 h of burn injury	12 (10 to 20) %	Six participants required repeat surgery to their burn wounds. Nine sessions overall were ceased due to pain > 7/10.
Schieffelers et al. [6]	Exercise = 29 Control = 29	**Duration:** 6 to 12 weeks. **Frequency:** 3 sessions per week. **Intensity:** In-bed exercises: 60% of peak MVIC, out-of-bed exercises: Determined using 8RM testing. 3 sets of 8 to 12 repetitions.	Yes—2 days a week, 24 min of interval training, 3 min bouts of 50 or 70% of peak wattage.	7 (5 to 9) days	60 (45 to 67.5) %	No exercise sessions were ceased due to pain. Four participants experienced postural hypotension transitioning from in to out of bed.
Schieffelers et al. [8]	Exercise = 28 Control = 29	**Duration:** Up to 8 weeks. **Frequency:** 3 sessions per week. **Intensity:** 60% of initial peak force produced during hand-held dynamometry or a three-repetition maximum test. Then adjusted weekly based on new peak force assessment. 8 to 12 repetitions.	Yes—2 days a week, 24 min of interval training, 3 min bouts of 50 or 70% of peak wattage.	0 (0 to 15) days	17 (12 to 32) %	One session was ceased due to an episode of vomiting. Main causes for incomplete or failed sessions were surgery or postsurgical immobilisation (60 sessions, 16 subjects), pain (44 sessions, 15 subjects), and uncooperative patient (13 sessions in 7 subjects).

**Table 2:** Practice recommendations for the prescription of resistance exercise in the acute adult burn setting.

Prescription Principles	Resistance Training
Frequency	3 times per week.
Intensity	60% of peak force from baseline assessment (assessed and updated weekly), with 3 sets of 8–15 repetitions per set.
Time	Continuing.
Volume	No established volume.
Progression	Should start with what they can accomplish on the day of initiation and then individualise, with daily progression based on objective measures (e.g., previous session RPE or 1–3RM or isometric strength assessment).
Major Considerations	Safety is the highest priority, so progression of load and intensity should be slow. Instruction of techniques should be provided by trained exercise professionals. Exercise prescription should be individualised based on the severity and location of burns, respecting daily pain levels and physical limitations. Hand burns may require specific adaptations to how patients interact with equipment (e.g., modified grip, assistive strapping, or alternative movement patterns).

## Data Availability

No new data were created or analysed in this study. Data sharing is not applicable to this article.
